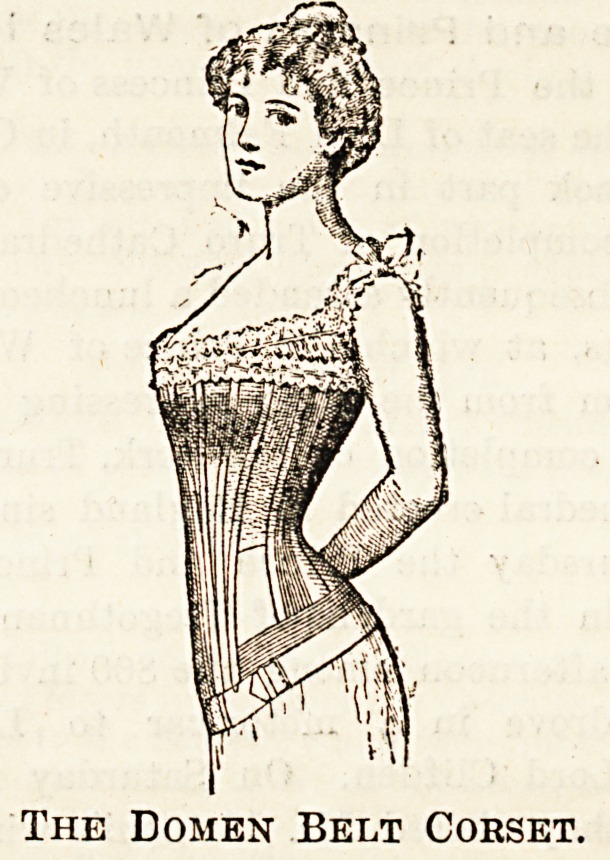# The Hospital. Nursing Section

**Published:** 1903-07-25

**Authors:** 


### The Hospital.

**Nursing Section.**

Contributions for this Section of "The Hospital" should be addressed to the Editor, "The Hospital" Nursing Section, 28 & 29 Southampton Street, Strand, London, W.C.

No. 878.—Vol. XXXIV. *SATURDAY, JULY 25, 1903.*

### Notes on News from the Nursing World.

### PRESENTATION OF MEDALS BY PRINCESS LOUISE.

An interesting function took place in connection with the inspection by the Princess Louise of the new hospital at Alton which bears her name. Her Royal Highness presented medals to the following nurses on the hospital staff:—Miss A. Garriock (matron), Miss L. W. Tulloh, Miss J. E. Dods, Miss A. F. Byers, Miss E. H. Hay, and Miss K. Ward, all of whom rendered valuable service during the South African war. Miss Garriock was superintendent for more than three years of No. 1 General Hospital, Wynberg; Miss Tulloh superintendent of the General Hospital, Bloemfontein and Norval Pont, for two years; Miss Dods a nursing sister for two years, and Miss Byers, Miss Hay, and Miss Ward were staff nurses for the same period.

### BELGRAVE HOSPITAL FOR CHILDREN.

The new Belgrave Hospital for Children in the Clapham Road, which was opened by Princess Henry of Battenberg on Monday, contains accommodation for twelve nurses. The dining-room, a lofty, well-lighted apartment of considerable size, is on the third floor, and leading out from it is a smaller room which is intended to be the nurses' sitting-room. Both these rooms are as yet unfurnished, but a member of the Committee of management, Mr. Percy Armytage, has generously undertaken to furnish both these. On the same floor are eight bedrooms, and on the top floor there are four cubicles. This is a blemish. There is no excuse for providing cubicles for the sleeping accommodation of either nurses or servants in a new building.

### THE ARMY NURSING SERVICE RESERVE.

There is an impression that the Army Nursing Service Reserve has been absorbed in Queen Alexandra's Imperial Military Nursing Service. This is a mistake. The Reserve not only exists, but is to be maintained in its original shape. Last week there was a meeting at 68 Victoria Street, at which several applications for membership were considered, and a few candidates were accepted. Although, of course, there is not nearly the rush there used to be during the South African War, the Service, we are informed, goes on adding to its members and is many hundreds strong. Queen Alexandra's Imperial Nursing Service takes the place of the Army Service only.

### LORD LEICESTER AND THE NURSING MOVEMENT.

In another part of the paper will be found a full account of the opening of the Nurses' Home in connection with the Norfolk and Norwich Hospital. It is entirely due to the munificence of the Earl of Leicester that the nurses are indebted for their new quarters, which will bear comparison with any similar institution in London or the provinces. Lord Leicester's benefactions to the hospitals in Norfolk are well known, but the latest instance of his generosity illustrates the great personal interest he takes in the welfare of nurses. For in addition to the £15,000, expended upon the erection and equipment of the home, he has given £5,000 in order, to quote his own letter which Lady Leicester read at the opening ceremony, "that the income of the hospital should not be heavily taxed to supply the necessary requirements of the nurses' home." That is to say, the interest on the £5,000 will suffice to meet the annual expenditure, and, instead of the board of management being in doubts as to their capacity to maintain the home in the future, they are in the happy position of knowing that for all time it is provided for. The part played by the Dean of Norwich in the matter is entitled to recognition. In December, 1896, he was the governor whose duty it was to inspect the hospital and to note his observations in the house visitors' book. In answer to the printed question, "Have you any observation to make?" Dr. Lefroy wrote, "Yes, the board of management should be prosecuted for allowing four nurses to sleep in one room. To escape condign chastisement they should either build a nurses' home or do the work themselves." It was largely in consequence of this entry that Lord Leicester offered to erect the home at his own expense. But for his liberality, the nurses of the Norfolk and Norwich Hospital might have had to wait a much longer period for adequate provision, and the conveniences and comforts they now possess would have been impossible.

### ST. FRANCIS HOSPITAL FOR INFANTS.

Following the formal opening last week of the St. Francis Hospital for Infants, in Denning Road, Hampstead, the whole of the nursing system will, we understand, be entirely reorganised at an early date. At present the staff consists of a matron, a head sister, and seven nurses. In each of the three wards one staff nurse is on duty in the day, and two nurses are on duty in the wards during the night. There are upwards of thirty in-patients and no out-patients.

### THE NURSES OF WESTMINSTER HOSPITAL.

In his report, for the fourth year, of an examination of the probationer nurses who are undergoing training at the nursing home in association with the Westminster Hospital, Dr. Hayward states that the examination, as on previous occasions, was made to consist of two parts—namely, a written examination on Elementary Anatomy and Physiology and on the general principles of Medical and Surgical Nursing; and a *viva-voce* examination of a quarter of an hour, in which each probationer was separately questioned, partly in subjects arising out of her answers to the written questions, and partly in other branches of the same subjects. He mentions that none of the 23 probationers who presented themselves for examination fell below the required minimum standard for passing of half the maximum number of marks obtainable. The first three names on the list in order of merit were Probationers Uglow, Featherstonehaugh, and Hannam, who obtained 75 per cent. and over the required maximum number of marks. Dr. Hayward considers that the results of the examination "reflect great credit on the matron, lecturers, and also the class-sisters, who must have taken infinite pains with their pupils, as well as the pupils themselves, who have worked hard to avail themselves of their opportunities"; and he adds: "While of necessity there must be personal and individual qualifications requisite for the making of a really good nurse which cannot be tested by any examination, it cannot but be that the possession of such knowledge and acquirements as the probationers have shown themselves to have must make them the better able to do credit to their training school as well as to themselves."

### TRAINED NURSES IN COUNTRY DISTRICTS.

IN the eighth annual report of the South Northamptonshire Nursing Club, Lady Knightley, the president, recalls the fact that when it was started there were many prophecies that it was hopeless to expect to raise sufficient money in a country district to support a highly-trained nurse. The main principles upon which the club was founded were that it should be worked on a provident basis, and that none but nurses with at least three years' training should be employed. The subscriptions were fixed at 4d. a month for working people, including their families, 10s. a year for farmers and tradesmen, and £1 for others, including their households. In the light of the results of eight years' experience, the predictions of failure are amusing. The club originally supplied one district with one nurse; it now supplies four districts of 22 parishes with four nurses. Moreover, it has paid its way unassisted by bazaars, sales of work, or any other form of charity. There was a small deficit last year, in consequence of extraordinary expenses, but this has been wiped out, and there is now a small balance in hand. The four nurses paid during the year 8,730 visits. Subscriptions amounted to £315, and midwifery fees to £28. On the other side salaries absorbed £277, and uniform £16. The remarkable success of Lady Knightley's steady adherence to the high standard of nursing in 22 country parishes in Northamptonshire, is a practical answer to the assertion, so often made, and acted upon, that in such districts the half-trained village nurse is a necessity.

### NURSING THE VICTIMS OF THE RAILWAY ACCIDENT.

THE sufferings of the numerous victims of the disastrous railway accident at Waterloo, near Liverpool, last week, were, as far as possible, mitigated by the prompt aid afforded by doctors and nurses. Soon after the calamity occurred a number of nurses hastened to the spot, and sought out the passengers who were maimed and helpless, affording very valuable and much appreciated help. Many of the injured were conveyed to the Bootle Borough Hospital, the nearest available, and there they received every attention from the matron, Miss Roberts, and her staff. Among the badly hurt in the hospital are a bank cashier whose face was terribly scalded, and a friend accompanying him who was injured about the legs, arms, and hands. The ticket collector at Waterloo Railway Station is also among the patients in the hospital. There are still thirteen cases under treatment, and we are glad to learn that they are progressing favourably.

### THE OFFICES OF THE CENTRAL MIDWIVES BOARD.

A MEETING of the Central Midwives Board was held at the Privy Council, Whitehall, last week, Dr. Champneys in the chair. The secretary reported that in accordance with the resolution of the Board, at the last meeting, he had taken the offices at No. 6 Suffolk Street, Pall Mall. The rooms were now being redecorated and it was hoped that the Board would be able to move in early in August. Dr. J. R. Kaye, County Medical Officer for the West Riding of Yorkshire, Dr. G. Reid, County Medical Officer for Staffordshire, and Dr. Shirley-Murphy, County Medical Officer for London, attended as a deputation from the Conference of County Medical Officers, in order to make certain suggestions with reference to the administration of the Act in county areas. At the conclusion of the discussion the chairman thanked the members of the deputation for their attendance, and for pointing out how certain difficulties in the administration of the Act might be overcome.

### ROYAL FREE HOSPITAL.

ON Monday the first sale supplementary to the recent bazaar at the Prince's Skating Club, Knightsbridge, in aid of the Royal Free Hospital took place at the Empress Room, Royal Palace Hotel, Kensington, which was lent for the occasion. At the bazaar about £3,500 was realised, and it is hoped, by means of supplementary sales, to raise the amount to £4,000. All the services in connection with the sale this week were given free of cost. The next sale will be held early in the spring at the house of Lady Llangattock, South Lodge, Rutland Gate.

### THE GRANARD SCANDAL.

THE suspension of Dr. Kenny by the Granard Board of Guardians has been removed by the Irish Local Government Board, and so far there is an improvement in the state of the affairs at the infirmary. The action of the Local Government Board proves beyond question that they are satisfied that the friction which occurred between the Guardians and the nuns on the one side, and Dr. Kenny on the other, as to the nursing, was not the fault of the latter. During Dr. Kenny's suspension and after the nuns, at the bidding of the Roman bishop of the diocese had left the infirmary, one of the lay nurses applied for medical aid for a patient and could not obtain it, though the master of the workhouse communicated with three doctors, the result being the death of the patient. We trust that, both in justice to the nurse, and also for the sake of future nurses, as well as patients, at the Granard Infirmary, this incident will be the subject of inquiry by the Local Government Board.

### BRENTFORD INFIRMARY NURSES.

The matron's report of the seventh year of the Training School at the Brentford Union Infirmary, Isleworth, contains some interesting details. Since July 1st, 1902, ten nurses who had completed their three years' engagement and been awarded training certificates left to take up various appointments. Eight probationers have been promoted to be staff nurses and one resigned after a long illness. Eleven new probationers entered; of these two left during the preliminary trial, one on account of health, the other owing to private family circumstances. There are now in the home 21 staff nurses and probationers. Two nurses who trained at the Infirmary a few years ago have returned as ward sisters. Dr. Fooks and Dr. Silvester have regularly given courses of lectures on physiology and anatomy as well as clinical instruction in the wards, and classes have been held by the matron and assistant-matron on nursing and bandaging. The fifth final examination for nurses in their third year was held by Dr. Seymour Sharkey, of St. Thomas's Hospital, on June 9th and 25th. Five nurses presented themselves for examination and all passed, Dr. Sharkey considering them "all good." He reports, "I have this day examined five nurses at the Infirmary and can congratulate those responsible for their teaching on the very marked proficiency they all showed." The names of those who passed are Marie Robinson, Florence E. Lowe, Charlotte E. Mellor, Agnes E. Williams, and Gertrude L. Souter. Massage classes have been regularly held, and all the nurses who went up for the examination held by the Incorporated Society of Trained Masseuses, London, passed successfully. Six nurses holding the Infirmary training-certificate obtained during the year appointments in general hospitals and infirmaries as ward sisters.

### A DANIEL COME TO JUDGMENT.

In "the humble opinion" of Mr. Charles Grayson, master of the Ipswich Workhouse, "there was no real necessity for a departmental committee on the question of workhouse nursing at the time it was appointed." Mr. Grayson, still maintaining his attitude of humility, also thinks that "the thing principally originated from the clamouring, selfish, and somewhat vindictive agitation of the Workhouse Nursing Association, and those they were able to persuade and incite by the plausible and exaggerated statements of facts, to their way of thinking." Hard words break no bones, and Mr. Grayson's imputation of motives to the members of the Workhouse Nursing Association will not do them any harm. But, as he suggests that the Local Government Board, having obtained the report of the departmental committee, will treat it as waste-paper, partly because they are fully occupied over matters of infinitely more importance and partly because so "many important Boards of Guardians" will not hear of altering the relative positions of the workhouse matron and the superintendent nurse, we hope that Mr. Walter Long will lose no time in making it clear that the exhaustive and expensive inquiry which he instituted was not intended, and is not going to be treated, as a hollow farce.

### PRIVATE NURSES AND WINE.

Complaint is made in a Bristol paper of the requirements of a private nurse who attended a middle-class family stricken with influenza. At supper, it is stated, the nurse, although she must have noticed in threadbare carpets and general shabbiness signs of a struggle to keep the home going, hinted that she took Burgundy with her meals, and in other ways made things uncomfortable. It is, we fear, impossible to prevent nurses who attach primary importance to their personal requirements from rendering the friends of their patients uncomfortable. But many of the best nursing institutions include in the conditions on which they send out members of their staff a provision that neither wine nor spirits shall be given to the nurses except by medical direction. This effectually prevents hints about Burgundy, and disposes of one question which, as in the case at Bristol, may otherwise cause irritation.

### THE FRIENDS OF ASYLUM PATIENTS.

A correspondent writes to point out that in not a few cases the friends of patients in asylums neglect their duty to their relatives. In her view, and in ours, it is unnatural for the friends of an insane person who has been received into a county lunatic asylum to ignore their plain duty to pay him regular visits, as and when permitted to do so by the authorities. Those authorities take infinite pains to encourage, under proper regulations, the visits of friends, and we are glad to know that in many instances the mental invalids are never forgotten by those at home. Our correspondent is under a misapprehension in supposing that the friends do not contribute to the maintenance of patients admitted to county lunatic asylums. The law provides that they shall so contribute, and the authorities are very careful to enforce these payments whenever the means of the families permit.

### NURSES' HOME AT GLOUCESTER.

A grand bazaar and garden fête will be held in the grounds of the Gloucester General Infirmary on Tuesday, Wednesday, and Thursdaay next week, in aid of the Nurses' Home Building Fund. The Duchess of Beaufort will perform the opening ceremony.

### A PUBLIC DEMONSTRATION OF PRACTICAL NURSING.

An interesting public demonstration of practical nursing was recently given in the amphitheatre of St. Luke's Hospital, New York. The programme was as follows:—1. Turning a mattress under a helpless patient. 2. Hot pack. 3 (*a*). Preparation for intravenous infusion of normal saline solution; (*b*) improvised ice-coil. 4 (*a*). Cupping, (*b*) mustard paste. 5. Mustard foot-bath. 6. Bandaging; (*a*) mastoid, modified Velpeau; (*b*) Barton, breast; (*c*) capeline, arm and shoulder spica. 7. Typhoid tub-bath. 8. Serving-tray. The typhoid tub-bath was given in a frame invented and perfected by Miss Ellicott, of the Johns Hopkins Training School for Nurses. For the preparation of a luncheon for a convalescent patient the time occupied was thirteen minutes. While the ice-cream was freezing an omelette was nicely browned, a salad prepared, a grape-fruit temptingly arranged, and a pot of tea made.

### The Nursing Outlook.

"From magnanimity, all fear above;
From nobler recompense, above applause,
Which owes to man's short outlook all its charm."

### THE RESPONSIBILITY OF THE NURSE.

There is nothing easier than to blame our circumstances, our parents, our teachers, and our pastors for all our own faults and follies. And it is just possible that the training schools are not solely responsible for the present disorganisation in the nursing world, but that the nurses themselves are failing somewhat in their larger professional duties. There are very few trades or professions completely in the hands of women, and in all public work women are in a minority. The result of the home-life that falls naturally to most women is that they are as a class unused to business methods, ignorant of how to organise, unaccustomed to public speaking or debate, and altogether too ready to dwell on the temporary and petty aspect of the case, instead of looking ahead, acting together, and securing the will of the majority. According to the last census there are 64,000 women nurses in England and Wales alone: they ought to be a power in the land, and to be capable of managing their own affairs, but as a matter of fact they are easily exploited by any woman who chooses to use them to force herself to the front, or by any body of managers who think that nursing is a charity and that women workers ought to do everything "for the love of God," and never consider wages. Far be it from us to teach that "nurses should first look after number one." We know no more mischievous and selfish policy, nor one that more deeply condemns its holder. It is because a petty personal view is so common that nursing as a profession does not take the position it should and is falling in public repute. Let a nurse remember always that she is a nurse, one of a great band of women fulfilling an honourable work; let it be her pride that nothing she does can ever bring discredit on her comrades; let her not think first of her own reputation, but of the reputation of her profession and of her sex.

It seems to us that this is where nurses fail chiefly at this juncture; they need to look forward and see whither they are being led and who are their leaders and whether the objects and aims are worth striving for, and if so, how they are each going to help. Every nurse should belong to some organisation, learn committee methods, have the courage to move amendments, and otherwise deal with those who would try to wrest nursing organisations out of the hands of nurses themselves. It is pitiful to hear a room full of nurses being scolded by a shrill-voiced "secretary" or "chair" who is no nurse and merely in a usurped position; and who runs a meeting entirely her own way simply because those present have not the courage of their opinions or the knowledge of how to present them. It is the most difficult thing possible to get any body of nurses to recognise, that the officials of their societies are their servants and not their masters, and that it is the members of the society who must insist on its policy being that they desire. Here are the holidays close upon us, and where is the conference that Princess Christian recommended the Royal British Nurses' Association to hold? We believe that the committee has met since the annual meeting, but has so far taken no action. If every nurse recognised herself as one unit of a vast body, and held herself responsible for the credit or discredit of that body, then it would be possible to push the necessary improvements of a growing profession.

Surely it is not necessary to start any new association of nurses? The multiplication of agencies is always to be regretted, and if only nurses would throw off their inertia, and recapture the societies now being run by outsiders, they might make their voices heard throughout the land, and the public would resume that confidence in the profession that is now being shattered because it is the wrong persons who speak, the non-nurses who agitate, the bad nurses who too often come to the front. Of course it is not to be expected that every nurse can speak in public, or serve on a committee, or write to the papers; in all these things knowledge and experience tell, and the amateur secretary, for instance, is generally a failure. But it is expected that nurses should begin to realise the power of the vote, the force that comes by united action, the control of its officials by the body of each society. It is expected that every nurse should realise her responsibility to do something to secure a more uniform standard of education and training for nurses, and the better organisation of the profession as a whole. There is no greater power than public opinion, and if this power were brought to bear to sweep the streets of flighty and disreputable women dressed as nurses; to force the smaller hospitals into combining together to secure more varied and lengthened training for their probationers; to insist on the larger training schools considering the needs of the public as a whole; to arrange for public examinations and a scheme of post-graduate teaching for the older private nurses; to demand the resignation of the assertive officials who generally dominate the nursing societies and the substitution for them of unselfish nurse-workers for the public weal, the impending crisis might be averted.

No profession ever rose more suddenly to a high and honourable position than that of nursing; no praise has been more lavish, or more deserved, than that bestowed during the last 50 years on women nurses. And England holds herself as a head of all other countries in this respect; and all the colonies know has been learnt from the mother country. The United States also has learnt all her nursing from us. It would be terrible indeed, then, if in England should come the first downfall in repute of this splendid work; and come it will if all the power is allowed to drift into the hands of self-centred assertive lay workers who grate upon all good taste; and if the real nurses, the true workers, the unselfish women, do not come forward and take their stand as those who alone have the right to represent the most noble profession on earth.

### Lectures on Ophthalmic Nursing.

By A. S. COBBLEDICK, M.D., B.S.Lond., Senior Clinical Assistant and late House-Surgeon and Registrar to the Royal Eye Hospital.

### LECTURE XV. — AFFECTIONS OF THE CORNEA, THEIR SYMPTOMS, DIAGNOSIS, AND TREATMENT.

SOME of the most troublesome corneal diseases, such as phlyctenular keratitis and pannus, have already been dealt with in previous lectures.

We now come to a disease which is, unfortunately, common in young patients whose parents have contracted syphilis in early life, viz., interstitial keratitis. It is most frequently met with between the ages of eight and fifteen years. Many of these cases show other signs of congenital syphilis, such as scarring around the mouth (especially at the angles), Hutchinson's teeth, *i e.*, notched central incisors, or auditory nerve deafness. In appearance the patients may look fairly healthy, and may show none of the afore-mentioned stigmata.

It is difficult to say what precipitates an attack. It may be that the changes consequent on the age of puberty, or the second dentition, act as exciting causes. Cases usually begin with pain and lachrymation. The infiltration of the cornea is first noticed at the margin. This slowly but surely increases, and is accompanied by engorgement of the blood-vessels in its neighbourhood. The cornea loses its clearness, and the surface, instead of being smooth, has the appearance of ground glass. The infiltration gradually spreads towards the centre of the cornea. It may be that the whole circumference of the cornea is affected, which means eventual involvement of the whole cornea.

Every degree of intensity is met with; in slight cases, a segment only of the cornea is affected, and this eventually clears so as to leave but little trace of the disease.

In more severe cases the whole cornea becomes involved; improvement is first noticed at the periphery, and continues towards the centre.

In very severe cases the whole cornea becomes blood red, or somewhat like a ripe cherry; the white of the eye is salmon pink in colour, and the condition of affairs is very serious as regards subsequent vision. As the redness passes off the cornea becomes quite white, but in the course of three or four months it is astonishing how much more the cornea clears than one might expect.

In severe cases the inflammatory process is not confined to the cornea, but involves the whole eye more or less, the iris and ciliary body are always inflamed, and the trouble may extend to the choroid coat or produce an optic neuritis.

Needless to say, the more acute the case the greater is the pain and photophobia, the patient's rest is broken, the appetite fails, and nutrition is lost.

### PROGNOSIS.

When a case is seen fairly early, it may be safely prophesied that the eye will get worse before any improvement takes place. It is also well to inform the child's parents that the other eye will probably become affected.

Except in slight cases, a guarded opinion should be given as to the impairment of vision that will result. Severe and most unpromising cases clear up wonderfully, but do not give distinct near vision.

### DIAGNOSIS.

As a rule this is not difficult; at times, however, in the early stages, there may be some doubt, especially when the congenital stigmata are absent. The general appearance of the patient, the ground-glass appearance of the cornea, and, when present, the salmon-pink patch at the periphery of the cornea, are enough on which to base the diagnosis.

### TREATMENT.

This must be constitutional as well as local.

*Local.*—As the inflammatory trouble often extends deeply the iris and ciliary muscle must be placed at rest by the use of atropine, either by ointment or drop (gr. iv. ad. ℥i.) used night and morning; this prevents any adhesion forming between the iris and lens. Rest must also be given by shielding the eyes from light either by the use of a large shade or a pair of large dark glasses.

Blisters and setons should not be used as they only add to the sufferer's misery.

*Constitutional.*—In the early stages mercury should be given in some form or other. The pulv. hydrarg. c̄. cretæ. at night time in gr. iii.—v. doses is perhaps the best.

As the acute stage passes, tonics, *e g*, syr. ferri. iodide. ʒi. t.d s p.c., and cod liver oil and malt must be given with a free diet, and as open-air a life as the patient's environment will admit.

If the cornea is left nebulous the yellow oxide of mercury ointment should be used night and morning, a small speck inserted into the conjunctival sac, and the part gently massaged for five minutes by moving the upper lid on the cornea.

If after the prolonged use of the ointment there is still considerable corneal opacity, gutt. adrenalin (1 in 3000) may be used three times a day, frequently with a most satisfactory result.

Some very bad cases of nebulous cornea have been treated by the *x*-rays with varying degrees of success; this treatment is still on its trial, and it is doubtful whether it will ever become a useful means of removing the opacity.

Of course, the most rational treatment for all these corneal troubles would seem to be their excision and implantation of a clear cornea, *e.g.*, a rabbit's: this has been practised in many cases, and the immediate result has seemed satisfactory. Eventually, however, the implanted portion became opaque and the condition of the patient unimproved.

Of the media of the eye which transmit light rays, the cornea is more frequently affected than any other; and, consequently, its affections are the chief cause, under this heading, of impaired vision. The lessons learnt are, never to ignore or treat lightly any affection of the cornea, and, in nearly all cases, to treat the original cause of the disease, which is usually constitutional, as well as the local manifestation.

### "The Hospital" Convalescent Fund.

THE Hon. Sec. begs to acknowledge, with thanks, the receipt of 5s. from the Travel Editor of THE HOSPITAL.

### Resignations.

THE matron of Dewsbury and District General Infirmary has resigned her appointment. Miss Nunn, who is leaving her post in order to be married, was trained at the Halifax Infirmary, and entered Dewsbury Infirmary as charge nurse. Nearly seven years ago she was promoted to the office of matron, which she has since filled to the satisfaction of the Board, the patients and the staff.

### Opening of the Leicester Nurses' Home of the Norfolk and Norwich Hospital.

BY OUR OWN CORRESPONDENT.

ON Thursday, July 16th, the new nurses' home in connection with the Norfolk and Norwich Hospital was formally opened by the Countess of Leicester, in the presence of a very large number of people.

The Home stands in spacious grounds on the site of an old-fashioned residence known as "The Shrubbery." This estate was purchased many years ago by the hospital board of management. For a considerable time, beyond yielding a certain income from the rental, it was of no particular use to the Hospital. But a few years ago the tenancy came to an end, and it was then thought advisable to use the old house for a nurses' home.

The nurses' accommodation being quite inadequate, some of the staff were for a time housed in "The Shrubbery." But it was soon found that the only satisfactory arrangement for the nurses would be to afford the whole of the staff suitable modern accommodation.

Lord Leicester came forward with a sum of £5,000 with which to start a subscription list for the conversion of "The Shrubbery" into a proper Nurses' Home. However, after very careful consideration the committee arrived at the conclusion that instead of altering and enlarging the old house it would be better to do away with it altogether and erect an entirely new home. Lord Leicester then added £10,000 to the £5,000 he had already promised, thus taking upon himself the entire financial burden.

### THE NEW HOME.

The new home is in the English Renaissance style, of red brick with stone dressings. It has four storeys. The principal entrance is on the side next the hospital and is approached by steps. There is a handsome tower, finished with an ogee roof.

The home contains 80 bedrooms, and these—with the exception of the night nurses' floor on the top, which is in white—are upholstered in green. Each room is 12 feet by 10 feet, and contains a hot-water radiator which can be controlled by the occupant of the room. There are two general sitting-rooms, one of which makes a delightful reading room, a sick-room, a sub-matron's private room, and a room for the night superintendent. The home is well furnished with bath-rooms, linen-rooms, and other conveniences.

### THE OPENING CEREMONY.

The Countess of Leicester, who wore a gown of soft grey satin, trimmed with lace and sequins, and a toque of black with red roses, was received at half-past two at Thorpe Station by the Chairman of the Board of Management (Colonel Dawson) and Mrs. Dawson, and by the vice-chairman (Dr. Beverley) and Mrs. Beverley. About 20 minutes later the party arrived at the hospital. A bouquet of pink carnations tied with a large chiffon bow was presented to the Countess by a little girl, Miriam Barrett, a patient in the children's ward.

A procession, headed by the civic mace-bearers, was then formed. The Town Clerk, the Mayor and the Countess, the Sheriff and the Mayoress, the deputy mayor, and Mrs. Copeman followed to the hall of the hospital, where the ceremony took place.

The Chaplain (Archdeacon Pelham) opened the proceedings with a special form of prayer. The following is one of the clauses:—

"O merciful Father, we beseech Thee to give Thy help and blessing to all who serve Thee in this hospital by ministering to the bodily and spiritual necessities of the sick. Enable them to do their work faithfully, as unto Thee and not as unto men; and grant that their labours may not be in vain. Hear us, O merciful Father, for Thy dear Son's sake, Jesus Christ our Lord. Amen."

### THE SPEECHES.

Colonel Dawson then explained how they had muddled along for twenty years without housing their nurses at all as they ought to be housed. Some few years ago he had to be there one morning and found a number of the nurses on the lawn behind the old quarters. When asked why they were there he was told that they were the nurses who had come off night duty and who had found their rooms so uncomfortable and so noisy that they were unable to get any rest in them. The Board of Management were helpless to remedy matters until Lord Leicester sent them his great cheque. He was glad to be able to state that the new Home would accommodate the private nurses as well as the hospital staff proper; and it was hoped and expected that the income brought in by the nurses who would be engaged in private nursing, and who would be housed there, would not only pay for the whole of the up-keep, but would realise a very handsome income besides to add to their funds.

Sir Charles Gilman, Chairman of the Building Committee of the home, then made a short statement with regard to the building and the generosity of Lord Leicester.

Colonel Dawson next presented Lady Leicester with a silver key as a memento of the occasion.

### LADY LEICESTER'S SPEECH.

The Countess in declaring the home open, said:—It has given me great pleasure to come to Norwich to-day. I feel that it is a very great honour to have been requested to perform the ceremony of opening the Leicester Nurses' Home. Lord Leicester wishes me to express his great regret at being unable to accompany me. Nothing would have given him greater pleasure or satisfaction than to see the successful completion of the work in which he has taken such a deep interest, and a work which he and I most earnestly wish may be for the lasting benefit of the county of Norfolk and the city of Norwich. Lord Leicester has given me a message, and he wishes me to bring it to you. It is this: "I am most anxious that the income of the hospital should not be heavily taxed to supply the necessary requirements of the nurses' home. I will therefore give £5,000 to be permanently invested, the interest therefrom to be given to meet the annual expenditure of the home. I wish this contribution to be a memorial to my old friend Mr. Cadge to whom the Norfolk and Norwich Hospital and the Cromer Convalescent Home are so deeply indebted." I have now much pleasure in declaring the Leicester Nurses' Home open.

### THE DAYS OF SAIREY GAMP.

The Mayor proposed "The thanks of the Governors to the Earl of Leicester for his bounty," and in doing so referred to his lordship's connection with the hospital, and his munificence.

The Dean of Norwich in seconding the resolution—which was supported by Dr. Beverley—referred to Sir Peter Eade's interesting history of the Norfolk and Norwich Hospital. In 1827 there was a resolution in the books of the hospital to the effect that any nurse selling beer to patients should be discharged. It seemed as if the Sairey Gamps of that day were really selling liquor without a license. In the very same year there was a nurse discharged for

### OPENING OF THE LEICESTER NURSES' HOME OF THE NORFOLK AND NORWICH HOSPITAL—*Cont*.

taking fees, administering improper food, allowing cards to be played and pipes to be smoked.

The Dean went on to speak of the higher demands which were now made by the public conscience regarding the Divine art of healing. He also related the experiences of a London surgeon who many years ago went to Norwich to perform an important surgical operation. He was selected not only on account of his especial experience in the particular operation, but mainly because he was at that time almost the only surgeon who carried out the details of the antiseptic system just introduced by Mr. (now Lord) Lister. "I remember well," said the Dean, "the condition he made in his letter to Mr. Cadge—'I must bring my own nurse. I can depend upon none you can supply.'" The Dean added, "I need scarcely observe that no such condition would now be made."

The speech-making concluded by the High Sheriff moving a vote of thanks to Lady Leicester for being present that day. The Sheriff of Norwich seconded, and Dr. Barrett supported the resolution.

Lady Leicester was then conducted by the Mayor over the home and back again into the hospital.

The wards were all decorated and one, carried out in mauve and white, the Countess noticed as being especially effective. The children's ward also looked very charming.

After the ceremony Mr. and Mrs. James Stuart entertained Lady Leicester to tea at Carrow Abbey. Among those asked to meet her were the lady superintendent and nursing sisters of the Norfolk and Norwich Hospital and the lady superintendent and nursing sisters of the Jenny Lind Infirmary.

### A Nursing Experience in Santa Catalina Island.

BY AN ENGLISH SISTER.

"I'VE a chance for you to get a splendid summer outing, combining business with pleasure. If you wish to take it you must be ready to start to-night." As a change of scene and air were what I had been longing for, but almost without hope, as the "state of the exchequer" was decidedly at low tide, my answer was a decided affirmative. When I found that my destination was to Santa Catalina Island, off the coast of California, a place I had read so much about, I was doubly pleased. I lost no time in packing, and was soon ready to accompany my patient, her husband, and friend.

### THE JOURNEY.

We had over three hundred miles to travel, but much of it was accomplished by night, which was bright moonlight. My patient, a pretty, plump little woman, whose looks certainly did not suggest that she was suffering from nervous prostration, refused to occupy a sleeper, as she was certain that we were doomed to destruction by the train going over a precipice, which seemed not impossible at the rate we went along the winding path through the mountains, so I began by having "a night of it." In the morning we reached Los Angeles, then changed for San Pedro, where we embarked for the island, which was not visible at the time. Although only 27 miles from land we had certain excitements, flying fish by the dozen, porpoises, and three whales, one of which kept so near the little steamer, that my patient clung desperately to me, fearing that the monster meant to upset us. However, nothing so sensational came to pass, else this article had never been written, for I am certain that my fate would have been to rest at the bottom of the Pacific Ocean, encircled by the arms of my patient, whose confidence in her nurse was only equalled in its intensity by her nervous terror. After two hours and a half on the boat, we landed at the wharf of Avalon, the beach town of the island. It even exceeded my expectations of beauty. The little town rises from the beach halfway up a mountain, the dwelling-places being on the high ground for the most part. All sorts and conditions of men, women, and children were there to meet the boat, and they were arrayed in all kinds and colours of costumes. Many were in bathing dresses which were very evidently never washed by "Father Neptune," but had been got up by fashionable laundries; others, however, were clad in ordinary, but neat costumes. There were seemingly all nations represented, Europeans—especially English and Americans—Negroes, Japanese, Chinese, Mexicans, Indians, etc., all talking at once.

### PICKLING THE PATIENT.

Whilst we rested and the gentleman of the party went to find us a house it was most amusing, and kept the mind of my patient well occupied, to watch the bathers pass and repass, and we wondered if we should ever get up courage to put on bathing suits and parade the town likewise; it seemed to me such a descent from the sublime to the ridiculous to change from my nursing uniform to the scanty attire for bathing. However, I knew it had to be done, as the doctor's orders were imperative to pickle my patient regularly every morning in "the briny," and not to encourage her in her fancy for long, trailing garments, nor for late dancing—in fact, to have her live as near to nature as possible in these ultra-civilised days. After a time my patient's husband, who is English like myself, returned to say that he had found a house or camp, so we started forward. We had to ascend part of the steep hill till we came to "The Terrace," up different tiers of which were placed camps, roofed-in tents, all more or less picturesque, most having quaint names. Ours was "Camp Sultana"; next door "Camp Rest Awhile"; near by twin tents had their signs up, "Ping Pong"; above us was "Do Drop In," also "Idle Days"; and the "Four Hungry" were near at hand. The furniture was as meagre as possible, and the cups, saucers, and plates, although antique, held out no temptation to the collector of curios, so substantial were they that I expect next century will find them still existing. But all the same we did enjoy our simple meals; they tasted well whether we got up early or late, and whether in full dress or bathing costumes we were always hungry. The swimming was perfect, the water being so buoyant I do not think we could have got drowned if we had tried. I was the only lady diver, but most could swim or float.

### A NOVEL ENTERTAINMENT.

One of our chief amusements was to be taken out in a glass-bottomed boat to see "the works of the Lord and his wonders in the deep." In this boat we could see over fifty feet down, and the scenery defied description, including rocky caverns of many colours, tenanted by curious looking sea monsters, while among the long waving beautiful varied seaweeds huge bright blue goldfish went sailing along, not a bit disturbed by our being overhead. Large abalone shells showed up their rainbow colours every now and then, and one almost longed to be a mermaid. The short sea trips were very interesting, one being to Moonstone Beach, where we were fortunate enough to pick up some nice specimens. Wild goats abound in the mountains, and we had the luck to see one and its kid not far distant. The seals seemed quite tame, we were able to get quite near to them. My patient improved only too quickly. The "recall" being sounded, I reluctantly had to return, after the most delightful holiday imaginable. Should any other nurses be offered the same opportunity, I strongly advise them to accept it, and hope that they will have an equally nice patient.

### Everybody's Opinion.

[Correspondence on all subjects is invited, but we cannot in any way be responsible for the opinions expressed by our correspondents. No communication can be entertained if the name and address of the correspondent are not given as a guarantee of good faith, but not necessarily for publication. All correspondents should write on one side of the paper only.]

### DISTRICT NURSING AT MATLOCK.

"Miss Margaret Harrison, President of the Matlock District Nurses' Association," writes from Dean Hill, Matlock: My attention has been called to a paragraph in your issue for June 20 in reference to the Matlock District Nursing Association and its affairs, in which the suggestion is made "that with proper economy in the management, and a little energy in collecting subscriptions," a second nurse might be provided. Permit me to say that the strictest economy is exercised over the funds of the Association. The nurse is paid according to the scale laid down by the Queen Victoria's Institute, comfortable but not expensive quarters being provided for her; and in order to save the Association's funds many of the necessary expenses (for appliances, food for the very poor, etc.) are met by private liberality. Moreover, the energy of the collectors is most admirable and praiseworthy. Every house in this large district is visited, and even the smallest contributions are carefully gathered in. It is due to the unfailing energy and enterprise of the ladies who undertake this work that we are able to supply our own district nurse. Probably the balance in hand at the beginning of the year may mislead outside people as to the financial condition of the Association, but the subscribers (for whom the report and balance sheet are prepared) need no explanation of it. They know that the current year's subscriptions are collected at the end of the previous year, so that we begin each year with our income in hand, an arrangement which is not common but which I think most people who are responsible for the working of charitable societies would pronounce to be excellent. We may sometimes have a second nurse in the district, but I think it might be left to those persons in this neighbourhood who have raised money for the one nurse to settle whether and when "the Association would be justified in making an addition to the staff."

[The report of the meeting on which our comments, of which Miss Harrison quotes the last sentence, were founded, did not disclose the interesting fact that the Matlock Nursing Association make it a point to begin each year with their income in hand. This makes all the difference.—Ed. "The Hospital."

### HOSPITAL ADMINISTRATION.

"E. H. B." writes: In these days when the question of reformed hospital administration is rife, a few words on the subject from a nurse's point of view may not be out of place. I would first point out what is already a patent fact to many, that in most administrative matters England is much behind the times. Looking a few years ahead I see—

1. England divided into sections, each section having its own general and isolation hospitals, with training schools for students and nurses attached, all under Government and supported by relative rates; the size, etc., of each hospital being determined by the population it has to accommodate.
2. A central Government Board in London controlling all, from which only examination papers and certificates would be issued, telephonic communication being established directly between it and each hospital.
3. Small ambulance-stations at different points in each section, connected by telephone, and a motor-ambulance system, with their respective hospitals. These to take the place of the present cottage hospitals and district nurses.
4. The abolition of private nursing homes, each hospital having one or more homes for paying patients connected with it, also a co-operative association for private nurses.
5. Compulsory payment of a small sum by all the hospital staff towards sick and old-age pension funds, the said sum to be deducted from the annual salaries for this purpose.
6. An eight-hours' day; superior, plentiful, and well-cooked food; nurses' quarters thoroughly well arranged, heated, and ventilated; and educated, gently-bred nurse-students, instead of the tired, over-worked "pros." (of all classes) too frequently met with to-day. In short, perfection!

### Novelties for Nurses.

**By Our Shopping Correspondent.**

### HYGIENIC CORSETS.

(Domen Belts Co., 456 Strand, London, W.C.)

Nurses will be glad to know that the straight-fronted belt corset made by the Domen Belts Company can now be obtained with short busks, which, besides being in accordance with present taste, are so much more comfortable than the long ones. The patterns just shown me by the manageress at 456 Strand are all considerably lower in the front than those formerly manufactured, while retaining all the hygienic advantages of the belt-corset. As is well known, this company some years ago produced, on medical advice, a combination of belt and straight-fronted corset with a view to preventing compression in the epigastric and umbilical region, and those who desire a shapely figure but wish also to avoid the horrors of tight-lacing, cannot fail to appreciate its advantages. Now that the short busk has been adopted the corset leaves nothing to be desired. There is a narrow elastic insertion at each side under the arms and the belt, passed round from front to back, is designed to prevent undue pressure downward, while giving support where it is needed. The corsets are made in white canvas for summer wear and this material is, of course, equally suitable for use in hot climates. Other materials

The Domen Belt Corset.

are coutille (white and grey), black drill, and natural-colour pure wool. I may add that the Domen Stoop-cure is an admirable corrective for round shoulders, and advise any who need external support to try it. It appears to be quite comfortable to wear, and the gentle reminder of a strong spring between the shoulder blades would certainly help in curing oneself of the habit of stooping. Nurses who want belts for patients should call and inspect the stock, or send for a catalogue by post.

### COOK'S TOILET SOAPS.

(E. Cook and Co., Limited, Soap Works, Bow, London, E.)

I have examined several samples of Cook's soaps and find them uniformly of a high standard of purity. They appear to be free from irritating substances, either of a mechanical or chemical nature, and are therefore eminently suitable for all who have delicate skins, and especially for nurses whose hands are liable to become rough and chapped from frequent contact with antiseptic solutions. The perfumes employed are delicate and well chosen.

### Echoes from the Outside World.

### The King and Queen in Ireland.

On Monday the King and Queen, accompanied by Princess Victoria, travelled from Euston Station to Holyhead on their way to Ireland, to pay their promised visit. There was a great crowd of spectators, not only at the London terminus, but also along the route from Buckingham Palace. Only one stop was made, at Crewe, in order to change engines, but at many of the stations people had assembled in order to cheer their Majesties as the train passed through. At Holyhead, on their arrival, they were presented with addresses from the County Council of Anglesey and the Holyhead Urban District Council, to both of which the King replied, observing that he was sure the Queen and himself would experience the pleasure anticipated during their visit to Ireland. They subsequently went on board the *Victoria and Albert*, which remained moored in the roadstead until four o'clock on Tuesday morning. The vessel containing the Royal party reached Kingstown at nine. Upon disembarking their Majesties were received by the Lord-Lieutenant. In reply to an address from the Kingston Urban Council, the King said that his visit fell at a time when bright hopes were entertained that a new era of peace had opened before Ireland, and it was his fervent prayer that these hopes might be realised. The Royal procession was then marshalled and departed on its way towards Dublin, the King and Queen being everywhere received with the utmost enthusiasm by the dense crowds who lined the route. In the evening the King gave a dinner party at the Viceregal Lodge.

### The Prince and Princess of Wales in Cornwall.

Last week the Prince and Princess of Wales arrived at Tregothnan, the seat of Lord Falmouth, in Cornwall, and on Wednesday took part in the impressive ceremony which marked the completion of Truro Cathedral. Their Royal Highnesses subsequently attended a luncheon in the Municipal Buildings, at which the Prince of Wales spoke and read a telegram from the King expressing his great satisfaction at the completion of the work, Truro being the first Anglican Cathedral erected in England since the Reformation. On Thursday the Prince and Princess attended a garden party in the gardens of Tregothnan and spent the whole of the afternoon among the 800 invited guests. On Friday they drove in a motor-car to Lanhydrock, the residence of Lord Clifden. On Saturday with their host and hostess they visited Mr. Marconi's wireless telegraph station at Poldhu, Mullion. At Helston and elsewhere along the route they were received with enthusiasm. The Prince of Wales ascended the staircase of the principal tower, 250 feet high. The Princess commenced the ascent, but after proceeding a short distance returned to the ground. During the afternoon two messages were received simultaneously from the wireless telegraph station at the Lizard, one offering respectful homage and welcome to the Prince of Wales, and the other a loyal message to the Princess. On Monday the Prince and Princess went to Falmouth to receive addresses of welcome and to lay the foundation stone of the new pier at Market Strand. They also visited the Royal Cornwall Hospital. Later in the day they proceeded to the banks of the Tamar to stay with Lord Mount Edgcumbe.

### Death of the Pope.

The Pope died at four minutes past four on Monday afternoon. At half-past eleven in the morning there was a marked change for the worse, and warning was immediately sent to all the Cardinals. The Pope's Sacristan then gave the absolution *in articulo mortis*. During the ceremony the Pope scarcely manifested signs of consciousness, but on its conclusion his three nephews entered the chamber, and as they knelt at the bedside he raised his hand in the act of blessing. Looking towards the Cardinals he repeated the gesture. The Pope preserved until the end the full use of his mental faculties. Born March 2nd, 1810, Leo XIII. was upwards of 93 years of age. He was the son of Count Pecci, who was once a colonel in the army of Napoleon the Great. His mother, a descendant of Cola di Rienzi, was one of those unpretending women who consecrate their lives to domestic duties and the succour of the poor and distressed. From her Vincent Joachim Pecci inherited the sweetness which, combined with strength, was a characteristic of his character. He was admitted to the subdiaconate and diaconate in 1837, became monsignor in 1838, Archbishop of Perugia in 1845—soon after a visit to England —Cardinal in 1854, Cardinal Chamberlain in 1877, and in July, 1878 he was elected Pope. Sympathetic messages with regard to the death of the Pope have been sent by King Edward and other heads of State. The body of the deceased Pontiff has been embalmed and laid in St. Peter's.

### Resignation of the Bishop of Manchester.

It was announced on Saturday that Dr. Moorhouse had resigned the see of Manchester, but that the resignation would not take effect until October 31st. It has since been intimated that the Bishop, instead of accepting the retiring allowance of one-third of the income, has decided to hand over the revenues of the see in full to his successor. Dr. Moorhouse, who is 77 years of age, was consecrated 27 years ago in October next to the Bishopric of Melbourne, having previously been vicar of St. John's, Fitzroy Square, and vicar of Paddington. While in Australia he was largely instrumental in obtaining the sum of £100,000 for the erection of a cathedral, and he was highly successful in the administration of the affairs of the diocese. In January 1886 he was appointed, at the instance of Lord Salisbury, to the Bishopric of Manchester, and returning to England was enthroned in May of the same year.

### A Famous Artist.

Mr. James McNeill Whistler died at his residence, Cheyne Walk, Chelsea, at the end of last week. Neither the exact date nor the exact locality of his birth are known, because he liked to wrap about his earlier years a veil of mystery, but it is believed that he was born in Massachusetts, U.S.A., about 1834. Some of his early years were spent in Russia, but when he was about 15 he returned to America and gained employment as a maker of maps and charts. Later, he found himself in Paris, studying in Gleyre's studio, where he met Du Maurier. Subsequently he came to London, and wrought the earliest of his famous etchings of the Thames. These he continued to produce during the sixties, and they are looked upon as some of the most perfect examples of the etcher's art. His "Venice" etchings also brought him much fame. Of portraits "Carlyle," "Sarasate," "Lady Archibald Campbell," and "My Mother" may be mentioned as being of note. In 1877 Ruskin published a criticism on a "Nocturne" exhibited by Mr. Whistler in the Grosvenor Gallery as "a pot of paint flung in the public face." This produced the well-known libel action, when Whistler obtained one farthing damages. Among his best known decorative works was the painting of the "Peacock Room" for Mr. Leyland, which was a truly marvellous scheme of colour. He was both witty and caustic, and "The Gentle Art of Making Enemies," his best known book, is most amusing reading.

### Appointments.

[No charge is made for announcements under this head, and we are always glad to receive, and publish, appointments. The information to insure accuracy should be sent from the nurses themselves, and we cannot undertake to correct official announcements which may happen to be inaccurate. It is essential that in all cases the school of training should be given.]

BOROUGH HOSPITAL, BIRKENHEAD.—Miss May R. Harrison has been appointed staff nurse. She was trained at the Lloyd Hospital, Bridlington, where she was afterwards staff nurse. She has also been district nurse at Nuneaton.

QUEEN CHARLOTTE'S LYING-IN HOSPITAL, LONDON.—Miss Caroline Atkins has been appointed superintendent of the out-patients' department. She was trained at Newport and Monmouthshire Hospital, and has since been staff nurse at Queen Charlotte's Lying-in Hospital.

ROYAL CORNWALL INFIRMARY, TRURO.—Miss E. Davies has been appointed matron. She was trained at the Royal Hospital, Portsmouth, and has since been staff nurse at the Children's Hospital, Moor Edge, Newcastle-on-Tyne; charge nurse at the North Devon Infirmary, Barnstaple; staff nurse at the Poplar Hospital for Accidents, London; sister at the Cumberland Infirmary, Carlisle; head sister at the County Hospital, Huntingdon; sister, night superintendent, and home sister at the Royal Infirmary, Bristol.

ROYAL HOSPITAL FOR SICK CHILDREN, EDINBURGH.—Miss Cordelia MacKay and Miss Marion Waugh have been appointed staff nurses. Miss MacKay was trained at the Royal United Hospital, Bath, and Miss Waugh at Dumfries and Galloway Royal Infirmary.

UCKFIELD COTTAGE HOSPITAL.—Miss Louisa Rich has been appointed matron. She was trained at the Warneford Hospital, Leamington, and has since held posts at Dawlish Cottage Hospital and Sherborne Hospital.

ULVERSTON UNION INFIRMARY.—Miss Hannah Mayoh has been appointed nurse in charge. She was trained at the Union Infirmary, Blackburn, where she has since been charge nurse.

### Presentations.

CITY HOSPITAL, BRADFORD.—On leaving the City Hospital, Bradford, where she has held the post of head nurse in the scarlet fever wards for nearly three years, Miss Dickinson was the recipient of a gold bangle, silk sunshade, and Russia leather purse, as tokens of respect and esteem.

### TRAVEL NOTES AND QUERIES.

ENGELBERG AND THE ITALIAN LAKES (Semloh).—Very glad to hear from you that the Pension was such a success. For Engelberg I do not remember what you wished to pay, but a very moderate place is Hôt-Pension Engel, terms from 6½ francs, or Hôt-Pension Hug, same terms. I cannot tell you the price of the ticket through the lakes because I am not in town, but send a stamped envelope to Messrs. Cook, Ludgate Circus, and ask them, it will not be much, for your route is really little more than over the St. Gothard, a lovely tour. At Locarno go to the Pension Belvedere, 5½ francs, this is on the road to the Madonna del Sass, or Pension Villa Muralto, 5 francs. I could not read the word but I think you mean *Lugano*. I should not advise a stay there, passing through is enough, go on to Como. It is difficult to decide where to stay, all is so lovely, but I incline to Bellagio Hôtel Pension des Etrangers, from 6½ francs. From there you can easily visit all the prettiest spots on the Lake. I think you should try to get 10 days for this Lake trip, a week makes it rather toilsome.

TRAVEL EDITOR.

### Notes and Queries.

**REGULATIONS.**

**The Editor is always willing to answer in this column, without any fee, all reasonable questions, as soon as possible.**

**But the following rules must be carefully observed :—**

1. **Every communication must be accompanied by the name and address of the writer.**
2. **The question must always bear upon nursing, directly or indirectly.**

**If an answer is required by letter a fee of half-a-crown must be enclosed with the note containing the inquiry.**

*Nursing in India.*

(161) *Mrs. W.* would be glad to know to whom she should apply for information concerning nursing in India.

Apply to the India Office, St. James's Park; also to the Up-Country Nursing Association, which furnishes information through Mrs Sheppard, 10 Chester Place, Regent's Park, N.W.

Can you help me to obtain posts in India for two nurses? *Nurse S.*

Write to the Secretary, the Colonial Nursing Association Imperial Institute, S.W.; and see answer to *Mrs. W.*

*Dispenser.*

(162) Will you tell me by what means a nurse can become a dispenser without attending classes in London?—*Nurse P.*

Write to the Secretary of the Pharmaceutical Society, 17 Bloomsbury Square, W.C.

*Salisbury Treatment.*

(163) Where is the nearest Nursing Home which undertakes the Salisbury treatment.—Edinburgh (*B. H.*).

We do not recommend private institutions. Almost all Nursing Homes receive patients for this special treatment.

*Nurses' Co-operation.*

(164) Will you kindly tell me if the London Association of Nurses is the same as the Nurses' Co-operation? If not, would you kindly send me the correct address of the latter? Would it be too much to ask for the names of a few of the best nursing associations?—*B. B.*

The address of the Nurses' Co-operation is 8 New Cavendish Street, W. That of the London Association of Nurses is 123 New Bond Street, W. You will find a list of associations managed by committees in "The Nursing Profession: How and Where to Train" (Scientific Press).

*Parish's Chemical Food.*

(165) Will you kindly tell me if Parish's Chemical Food can be obtained in capsules or tabloids? If it is, is the preparation equally good?—*M. A. F.*

Inquire at your local chemist's.

*Lepers.*

(166) Can you tell me to whom I ought to apply for information as to the nursing of lepers in India or the Colonies?—*H. L.*

For India, apply to the Secretary and Superintendent, Mission to Lepers in India and the East, 17 Greenhill Place, Edinburgh. For the Colonies, to the authorities at the different leper settlements. See "Burdett's Hospitals and Charities" for lists.

*The Royal National Pension Fund.*

(167) Are nurses who are illegitimate, say, through an illegal marriage eligible for the Pension Fund? What is the British Nurses' Association?—*Ignoramus.*

A nurse of good character is eligible for the benefits of the Royal National Pension Fund for Nurses, whatever her parentage may have been. The Royal British Nurses' Association is an association of trained nurses for mutual advantage.

*Electrical Treatment.*

(168) Can you kindly tell me where a young man could be received for electrical treatment where the latest and best methods could be obtained. He would need a private room, etc.—*Spero.*

Advertise for what you require.

*America.*

(169) 1. Will you kindly tell me the names and addresses of the best hospitals, nursing institutions and district nursing associations in New York City? 2. Are there openings for thoroughly trained nurses there? 3. What would a private nurse's salary be per week? 4. Is it possible to get an introduction to a doctor who would employ a trained nurse there? 5. Would the L.O.S. enable a nurse to practise among the poorer classes and receive her own fees?—*N. F. G.*

1. See list in "The Nursing Profession; How and Where to

Train." 2. America has plenty of nurses of her own. 3. It depends upon circumstances. 4. Only privately. 5. Yes.

*Notice.*

(170) Will you kindly tell me what notice a nurse is supposed to give in a private house on her own account, no previous arrangement having been made in this respect?—*Sister B.*

If weekly payments are made, then a week's notice will be required; if monthly, a month's notice; if the engagement is by the year, then a quarter's notice is generally given.

*Home.*

(171) Will you kindly tell me of a nursing home or pension at Hyeres, or Cannes, suitable for a lady suffering from chronic bronchitis?—*M. G.*

The Lady Superintendent, the Nice Nursing Institute, Villa Pilatte, Avenue Desambrois, Nice; and the English Nurses' Institute, Sunny Bank, Via Borgo Pescio, San Remo, might be able to help you.

*Abroad.*

(172) Will you kindly advise me as to nursing abroad? I am fully-qualified nurse anxious to go to one of our Colonies. I should prefer new Zealand, but I am ready to go to any one which is most suitable—*Anxious to go Abroad.*

You might apply to the Colonial Nursing Association, Imperial Institute, S.W.

*Feeble Intellect.*

(173) Will you kindly tell me if there is in London a home or institute where those of feeble intellect, but not insane, are cared for?—*Sister Edith.*

Apply to the National Association for Promoting the Welfare of the Feeble-minded, 53 Victoria Street, S.W.

*South Africa.*

(174) Will you kindly tell me if a nurse wishing to take a post in South Africa could get any help towards the expense of the voyage?—*A. C.*

The hospital making an engagement will often help with passage money. By advertisement you might hear of some one going out who would give you passage in exchange for services.

*Hospital Training.*

(175) Will you kindly tell me if there is a hospital for consumption in Herefordshire, and also say if probationers without any previous training are trained in nursing consumption only, in the open-air sanatoria?—*M. P.*

There is no special hospital for consumption in Herefordshire. You should train first at one of the large hospitals for consumption.

*M.D.*

(176) 1. Can you kindly give me any advice as to my studying to become an M.D.? 2. Would it be possible to do hospital nursing at the same time?—*A. C.*

1. Write to the Secretary, the London School of Medicine for Women, 8 Hunter Street, Brunswick Square, W.C. 2. No.

*Maternity Training Schools.*

(177) Please name two or three good maternity training schools. Is Queen Charlotte's the best?—*L. M. H.*

Queen Charlotte's is the largest, but excellent training may also be obtained at the British Lying-in Hospital, Endell Street, St. Giles, W.C.; the City of London Lying-in Hospital, City Road, E.C.; the Clapham Maternity Hospital, Jeffreys Road, Clapham, S.W.; the East End Mothers' Home, Commercial Road, E.; and the General Lying-in Hospital, York Road, Lambeth, S.E.

*Sanitary Inspector.*

(178) I am a trained nurse and am anxious to learn all about the work of a lady sanitary inspector.—*Mavourneen.*

Full particulars may be obtained by writing to the Hon. Secretary, the Sanitary Inspectors' Examination Board, 1 Adelaide Buildings, London Bridge, E.C.

*Maternity Certificate.*

(179) I have been in my present situation as district nurse and midwife for two years. How am I to get a midwife's certificate?—*J. K.*

Write to the Secretary of the Midwives' Institute, 12 Buckingham Street, Strand, W.C.

**Standard Nursing Manuals.**

"The Nursing Profession: How and Where to Train." 2s. net; 2s. 4d. post free.

"Nursing: Its Theory and Practice." (Revised Edition). 3s. 6d. post free.

"Surgical Ward Work and Nursing." (Revised Edition). 3s. 6d. net.; 3s. 10d. post free.

"Practical Handbook of Midwifery." (New Edition). 6s. net; 6s. 3d. post free.

"Notes on Pharmacy and Dispensing for Nurses." 1s. post free.

"Fevers and Infectious Diseases." 1s. post free.

"The Art of Massage." (New Edition). 6s. post free.

### For Reading to the Sick.

### "THOU VISITEST THE EARTH AND BLESSEST IT."

All this world is God's own field,
Fruit unto His praise to yield:
Wheat and tares therein are sown,
Unto joy or sorrow grown:
Ripening with a wondrous power
Till the final Harvest-hour:
Grant, O Lord of life, that we
Holy grain and pure may be.

For we know that Thou wilt come,
And wilt take Thy people home;
From Thy field wilt purge away
All that doth offend, that day:
And Thine Angels charge at last
In the fire the tares to cast,
But the fruitful ears to store
In Thy garner evermore.

*Henry Alford.*

Have you ever looked from your window across the road and over the thorn hedge, on to a field lately reaped? How bare and flat it seems; the waving corn and its golden glory is all gone, and in its place is a dull, monotonous, yellow-brown stretch of stubble. But go forth from your room, walk into that field, and look down. Is it bare and monotonous? Myriads and myriads of tiny flowers, some in bud and some in bloom, are springing up everywhere between the stubble stalks: some are clinging round the stones, some are covering the brown earth, others are pushing their way through the intricate tangle of grass and weeds; but all are eagerly stretching forwards and upwards to greet their great benefactor the sun, whose home lies so many millions of miles away. There they are in their lowly profusion a perfect wealth of beauty to eyes which love them. Even the heedless wayfarer is conscious of their presence, for as he treads them under foot the air is laden with their delicate fragrance. Even so is the Christian mourner's life. To a careless eye the loss is all that is known, but from the broken heart and the crushed spirit will arise the lovely flowers and fruit which mark the child of God.—*Mrs. C. G. Campbell.*

Sweet nurslings of the vernal skies,
Bathed in soft airs, and fed with dew,
What more than magic in you lies,
To fill the heart's fond view?
In childhood's sports, companions gay,
In sorrow, on life's downward way,
How soothing! in our last decay
Memorials prompt and true.

Ye dwell beside our paths and homes,
Our paths of sin, our homes of sorrow,
And guilty man, where'er he roams,
Your innocent mirth may borrow.
The birds of air before us fleet,
They cannot brook our shame to meet—
But we may taste your solace sweet
And come again to-morrow.

## Figures and Tables

**Figure f1:**